# 

**Published:** 1898-01

**Authors:** 


					﻿Vol. XVIII.
JANUARY, 1898.
NO. 1.
THE
JIOMCOPATHIC pHY^lClAfl,
A Monthly Journal of Homoeopathic Materia Medica
and Clinical Medicine.	<7
" He u a freeman whom truth makee free, and all are elavet betide."
“Seek the Truth: come whence it may, coet what it will."
BDITED AND PUBLISHED BY
WALTER M. JAMES, M. D.
PHILADELPHIA
nsro. 1231 LOCUST STREET^
X»Q JKT 30.0 29 ;
ALFRED HEATH At CO., Sole Agents fob Gbkat Britain,
114 Ebury Street, Eaton Square.
4®“Fearir/ Subscription, $2.50, invariably in advance. Single member e, 25 eU.
Entered at the Philadelphia Poet-OSee u Beoon .-Claei Mall Matter.
				

